# Real-Time Surveillance for Respiratory Disease Outbreaks, Ontario, Canada

**DOI:** 10.3201/eid1505.081174

**Published:** 2009-05

**Authors:** Adam van Dijk, Jeff Aramini, Graham Edge, Kieran M. Moore

**Affiliations:** Queen’s University, Kingston, Ontario, Canada

**Keywords:** Respiratory infections, surveillance, disease outbreaks, emergency departments, Canada, dispatch

## Abstract

To validate the utility of a chief complaint–based emergency department surveillance system, we compared it with respiratory diagnostic data and calls to Telehealth Ontario about respiratory disease. This local syndromic surveillance system accurately monitored status of respiratory diseases in the community and contributed to early detection of respiratory disease outbreaks.

The threat of emerging infectious diseases, such as severe acute respiratory syndrome and pandemic influenza, makes early detection of health events critical for effective control and intervention of such outbreaks. By using alternative, electronic data sources (*1*), real-time syndromic surveillance systems have the potential to detect outbreaks of respiratory disease before conventional diagnosis- and laboratory-based surveillance identifies them. Many of these alternative data, such as sales of over-the-counter drugs, telephone health hotlines, and emergency department (ED) triage data, already are routinely collected.

ED syndromic surveillance typically uses a patient’s chief complaint (CC) as recorded by a triage nurse. Respiratory CC data correlate strongly with discharge data (*2*). CC data from EDs have been successfully integrated into several surveillance systems (*3**,**4*), including the ED system in Kingston, Ontario, Canada (*5**,**6*). CC data are effective for early identification of influenza outbreaks (*7**,**8*).

In September 2004, the Queen’s University Emergency Syndromic Surveillance Teama created a real-time emergency department surveillance system (EDSS) to monitor respiratory and gastrointestinal CCs temporally and spatially. Seven hospitals from the southeastern Ontario region fed electronic CC data into the system. Routine patient data collected without identifiers include date and time of visit, demographic information, 5-digit postal code of residence, Canadian Triage Acuity Score, and CC or reason for visit. The National Ambulatory Care Reporting System (NACRS) gathers data for hospital- and community-based ambulatory care, day surgery, outpatient clinics, and EDs. Records contain patient diagnosis according to International Classification of Diseases, 10th Revision–Canadian Enhancement (ICD-10-CA) codes. Every hospital in Ontario submits data to NACRS, and the system includes demographic, clinical, and administrative data for the entire province (*9*). Telehealth Ontario is the teletriage helpline available free to all Ontario residents 24 hours a day, 7 days a week. Callers are connected to skilled nurses who assess symptoms over the phone and assist callers in making the most appropriate healthcare decision (*10*). Each caller’s demographics are recorded, and each call is assigned a guideline after the nurse has gone through a list of questions about why the person has called.

## The Study

We conducted an investigation to verify and validate the utility of a triage CC-based EDSS in southeastern Ontario as a tool for monitoring respiratory disease by comparing it retrospectively with data from NACRS and Telehealth Ontario. This study was part of a broader research project approved by the Queen’s Research Ethics Board and adheres to the principles and policies for the protection of personal health information charter.

We retrospectively studied data for July 4, 2004–March 31, 2006. Daily counts of discharges of persons with respiratory disease based on ICD-10-CA codes were obtained from the NACRS database, and counts of respiratory CCs were likewise collected from EDSS. Patient location was determined by forward sortation address (i.e., first 3 digits of postal code) and health unit code in the NACRS database and by specific reporting hospital in the EDSS data set. Weekly Telehealth Ontario counts of respiratory disease based on guidelines were also obtained. We categorized Telehealth Ontario calls into episodes of upper or lower respiratory disease on the basis of a priori classification schemes verified by other research (*6**,**11**–**13*). Telehealth Ontario calls were geolocated by forward sortation address. All data were compiled into weekly totals (Sunday–Saturday) from each of the 3 nonidentifiable data sets.

During July 2004–March 2006, EDSS contained 29,668 reports of respiratory diseases in persons seeking care at 1 of the 7 area hospitals. During the same period, Telehealth Ontario received 4,247 calls about upper and lower respiratory disease, and NACRS recorded 19,315 cases of respiratory disease from southeastern Ontario. Analysis comparing the EDSS respiratory CCs with the Telehealth Ontario calls about respiratory disease (Figure) resulted in a Spearman correlation coefficient of 0.91, indicating good correlation. Analysis comparing the EDSS respiratory CC to all NACRS respiratory disease diagnoses resulted in a Spearman correlation coefficient of 0.98, indicating very good correlation. All correlations were highly significant (p<0.0001). Correlations were highest and most significant when no time lags were included in the models.

**Figure:**
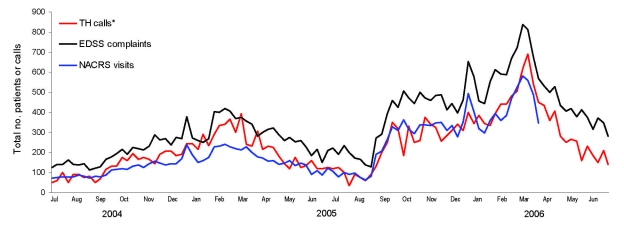
Weekly totals of emergency department surveillance system (EDSS) respiratory chief complaints, National Ambulatory Care Reporting System (NACRS) respiratory visits, and calls to Telehealth Ontario (TH) about respiratory illness, Ontario, Canada, July 2004–June 2006.

## Conclusions

This study verified that ED CC data can be used as a timely source of surveillance for respiratory diseases. ED CCs in southeastern Ontario strongly correlated in time with NACRS respiratory discharge diagnoses and calls to Telehealth Ontario about respiratory disease. NACRS data are unavailable to public health stakeholders in a timely enough fashion to be useful for day-to-day monitoring of respiratory disease trends in the community, whereas ED CCs are available electronically and in real time.

As expected, EDSS CCs about respiratory disease peaked during the influenza seasons (early November 2004–mid-April 2005 and early December 2005–early May 2006) (Figure); however, EDSS CCs continued to fluctuate during the influenza off season, probably because of other respiratory pathogens. The nature of the EDSS data did not allow us to separate respiratory complaints related to influenza from those related to other pathogens. Further analysis is needed to correlate CC subsets with laboratory-positive influenza cases.

One consideration for interpreting EDSS data is that the hospitals in this surveillance system include large referral hospitals that have high volumes of patients visiting from outside our health unit boundaries. Although this large referral area may result in higher counts of visits than reflected in the NACRS database, we believe it does not affect our interpretation of the data. Even though each hospital is required to submit data, it is still possible that not all records from each hospital are sent on time to NACRS.

Although the public health system has accepted syndromic surveillance as a useful tool, doubts remain about its anticipated early warning benefits (*14*). These potential benefits cannot be tested because no large-scale outbreaks have occurred since the inception of our real-time syndromic surveillance system. Protocols of many pandemic-preparedness plans include monitoring of real-time systems. When a pandemic occurs, syndromic surveillance may be able to help healthcare workers recognize a potential outbreak, which theoretically could help them mitigate effects on society earlier. Our study demonstrates that in southeastern Ontario, ED CCs accurately reflect respiratory conditions of patients in the area. The correlations found strongly suggest that EDSS accurately monitors respiratory disease in the community and contributes to early detection of respiratory disease outbreaks. We continue to monitor the system from day to day and have increased the number of reporting hospitals to 9. Future studies will use laboratory data to assess the value of advanced warning on a number of syndromes captured in our system.

## References

[1] Rolland E, Moore KM, Robinson VA, McGuinness D. Using Ontario's “Telehealth” health telephone helpline as an early-warning system: a study protocol. BMC Health Serv Res. 2006;6:10. 10.1186/1472-6963-6-10 16480500 PMC1431529

[2] Begier EM, Sockwell D, Branch LM, Davies-Cole JO, Jones LH, Edwards L, The national capitol region’s emergency department syndromic surveillance system: do chief complaint and discharge diagnosis yield different results? Emerg Infect Dis. 2003;9:393–6. 12643841 10.3201/eid0903.020363 PMC2958546

[3] Jorm LR, Thackway SV, Churches TR, Hills MW. Watching the games: public health surveillance for the Sydney 2000 Olympic Games. J Epidemiol Community Health. 2003;57:102–8. 10.1136/jech.57.2.102 12540684 PMC1732372

[4] Gesteland PH, Gardner RM, Tsui FC, Espino JU, Rolfs RT, James BC, Automated syndromic surveillance for the 2002 Winter Olympics. J Am Med Inform Assoc. 2003;10:547–54. 10.1197/jamia.M1352 12925547 PMC264432

[5] Espino JU, Wagner MM, Szczepaniak C, Tsui F-C, Su H, Olszewski R, Removing a barrier to computer-based outbreak and disease surveillance—the RODS open source project. MMWR Morb Mortal Wkly Rep. 2004;53(suppl):32–9. 15714624

[6] Lombardo J, Burkom HS, Elbert E, Magruder SF, Lewis S, Loschen W, A systems overview of the Electronic Surveillance System for the Early Notification of Community-Based Epidemics (ESSENCE II). J Urban Health. 2003;80(Suppl 1):i32–42. 12791777 10.1007/PL00022313 PMC3456555

[7] Irvin CB, Nouhan PP, Rice K. Syndromic analysis of computerized emergency department patients’ chief complaints: an opportunity for bioterrorism and influenza surveillance. Am Emerg Med. 2003;41:447–52. 10.1067/mem.2003.104 12658241

[8] Muscatello DJ, Churches T, Kaldor J, Zheng W, Chiu C, Correll P, An automated, broad-based, near real-time public health surveillance system using presentations to hospital emergency departments in New South Wales, Australia. BMC Public Health. 2005;5:141. 10.1186/1471-2458-5-141 16372902 PMC1361771

[9] Canadian Institute for Health Information. Executive summary: database background and general data limitations documentation. National Ambulatory Care Reporting System (NACRS) FY 2005–2006. Ottawa (Ontario, Canada): The Institute; 2006 [cited 2008 Mar 10]. Available from http://secure.cihi.ca/cihiweb/en/downloads/NACRS_Background_General_Data_Limit_Executive_Summary_2005-2006_e.pdf

[10] van Dijk A, McGuinness D, Rolland E, Moore KM. Can Telehealth respiratory call volume be used as a proxy for emergency department respiratory visit surveillance by public health? CJEM. 2008;10:18–24. 18226314 10.1017/s1481803500009969

[11] Tsui F-C, Espino JU, Dato VM, Gesteland PH, Hutman J, Wagner MM. Technical description of RODS: a real-time public health surveillance system. J Am Med Inform Assoc. 2003;10:399–408. 10.1197/jamia.M1345 12807803 PMC212776

[12] Cooper DL, Smith GE, Hollyoak VA, Joseph CA, Jones LH, Chaloner R. Use of NHS direct calls for surveillance of influenza—a second year’s experience. Commun Dis Public Health. 2002;5:127–31. 12166298

[13] Harcourt SE, Smith GE, Hollyoak V, Joseph CA, Chaloner R, Rehnman Y, Can calls to NHS Direct be used for syndromic surveillance? Commun Dis Public Health. 2001;4:178–82. 11732356

[14] Zheng W, Aitken R, Muscatello DJ, Churches TR. Potential for early warning of viral influenza activity in the community by monitoring clinical diagnoses of influenza in hospital emergency departments. BMC Public Health. 2007;7:250. 10.1186/1471-2458-7-250 17877836 PMC2075512

